# Hospital admissions and deaths due to acute cardiovascular events during the COVID-19 pandemic in residents of long-term care facilities

**DOI:** 10.1038/s41598-023-35816-y

**Published:** 2023-05-26

**Authors:** Paul Gellert, Raphael Kohl, Kathrin Jürchott, Betty Noack, Christian Hering, Annabell Gangnus, Elisabeth Steinhagen-Thiessen, Wolfram J. Herrmann, Adelheid Kuhlmey, Antje Schwinger

**Affiliations:** 1 Institute of Medical Sociology and Rehabilitation Science, Charité – Universitätsmedizin Berlin, Charitéplatz 1, 10117 Berlin, Germany; 2 WIdO – AOK Research Institute, Berlin, Germany; 3 Department of Endocrinology, Diabetes and Metabolism, Charité – Universitätsmedizin Berlin, Berlin, Germany; 4 Institute of General Practice and Family Medicine, Charité – Universitätsmedizin Berlin, Berlin, Germany

**Keywords:** Health services, Public health

## Abstract

Hospital admissions due to acute cardiovascular events dropped during the COVID-19 pandemic in the general population; however, evidence for residents of long-term care facilities (LTCF) is sparse. We investigated rates of hospital admissions and deaths due to myocardial infarction (MI) and stroke in LTCF residents during the pandemic. Our nationwide cohort study used claims data. The sample comprised 1,140,139 AOK-ensured LTCF residents over 60 years of age (68.6% women; age 85.3 ± 8.5 years) from the largest statutory health insurance in Germany (AOK), which is not representative for all LTCF residents. We included MI and stroke admission and compared numbers of in-hospital deaths from January 2020 to end of April 2021 (i.e., during the first three waves of the pandemic) with the number of incidences in 2015–2019. To estimate incidence risk ratios (IRR), adjusted Poisson regression analyses were applied. During the observation period (2015–2021), there were 19,196 MI and 73,953 stroke admissions. MI admissions declined in the pandemic phase by 22.5% (IRR = 0.68 [CI 0.65–0.72]) compared to previous years. This decline was slightly more pronounced for NSTEMI than for STEMI. MI fatality risks remained comparable across years (IRR = 0.97 [CI95% 0.92–1.02]). Stroke admissions dropped by 15.1% (IRR = 0.75 [CI95% 0.72–0.78]) in the pandemic. There was an elevated case fatality risk for haemorrhagic stroke (IRR = 1.09 [CI95% 1.03–1.15]) but not for other stroke subtypes compared to previous years. This study provides first evidence of declines in MI and stroke admissions and in-hospital deaths among LTCF residents during the pandemic. The figures are alarming given the acute nature of the conditions and the vulnerability of the residents.

## Introduction

Healthcare utilization during the pandemic including in-hospital care decreased massively in most countries around the globe (e.g.,^1–6^). Rates of emergency hospital admissions due to acute events dropped in the general population, including cardiovascular diseases (CVD) such as myocardial infarction (MI) and stroke (e.g.,^7–10^). Data from nine hospitals in England and Scotland during the first lockdown until May 2020 showed substantial reductions in total hospital (58%) and emergency department (53%) admissions compared to the previous years (CVD-COVID-UK Consortium;^11^). In a meta-analysis that included 27 studies published before August 12, 2020, eleven studies found a decrease in admissions due to acute coronary events (between 40 and 50%) and five studies showed a decrease in stroke admissions (between 12 and 40%). For MI subtypes, four studies showed a greater decrease in non-ST-segment elevation myocardial infarctions (NSTEMI) compared to ST-segment elevation myocardial infarctions (STEMI). At the same time, there was one study that did not find differences between NSTEMI and STEMI^8^. Finally, a recent German study found a decline in MI rates by 44% during lockdown in both STEMI and NSTEMI patients, which was present independently of gender and age^12^. Concerning CVD mortality, Kiss et al. found in their meta-analysis four studies that reported higher mortality rates during the pandemic, where the increase in mortality rates ranged from 4.1 to 9.6%, compared to earlier years^8^.

Residents of long-term care facilities (LTCF) not only faced high mortality and hospitalization rates due to COVID-19^1^; they may further be subject of greatly decreased hospitalization rates for CVD compared with rates prior to the pandemic. There is evidence from Ontario, Canada that this was true for older adults including community-dwelling older adults with dementia^13^, but evidence for LTCF residents is rare. An exception constitutes data from England that indicate a substantial decrease during the first wave of the pandemic from March until June 2020, with emergency admissions for those from nursing homes dropping by 29% for acute coronary syndromes and by 25% for stroke^14^. Whether this figure can be reproduced with more substantial data from other countries remains unknown.

Our primary aim was to investigate the rates of hospital admissions due to MI and stroke in LTCF residents during the COVID-19 pandemic. Our assumption was that the rate of hospital admissions due to acute cardiovascular events was lower compared to the last five years before the pandemic. Furthermore, we investigated the number of deaths due to MI and stroke, as well as case fatality rates of LTCF residents during the pandemic, compared with previous years.

## Methods

The present cohort study draws on claims data of the largest German statutory health and long-term care insurance fund group (AOK). The AOK has a market share of 49% among residents of LTCFs in Germany, thus is not representative for the German population of LTCF residents. Our study includes all AOK insured people over 60 living in LTCF during the study period. Data on CVD-related hospital admissions and deaths among residents from the time of the first three waves of the pandemic (January 2020 to end of April 2021) were compared with the five years before (2015–2019). The study was part of the COVID-Heim project, which was funded by the National Association of Statutory Health Insurance Funds. All methods were carried out in accordance with relevant guidelines and regulations including the Declaration of Helsinki. Ethical approval was received by the local ethics committee of the Charité (EA1/299/20). Informed consent was not obtained as routine data from health and care insurance funds are anonymous social data (according to the German law §67 Abs. 1 SGB X) and consent is not required nor possible. The need for informed consent was waived by the ethics committee of the Charité since anonymous social data was used. The AOK routine data are available in the Scientific Institute of the AOK (WIdO) without direct personal references and are treated as research data in accordance with the requirements of Article 89 of the General Data Protection Regulation of the European Union (EU GDPR). Due to data protection regulations, data cannot made available, without providing a formal application at WIdO.

### Measures

CVD hospital admissions and in-hospital deaths of AOK-ensured LTCF residents were counted based on valid and discharge ICD-10-based hospital diagnoses. Hospital admissions with a transfer direct transfer were counted as one. Admissions for acute MI were subdivided into STEMI (ICD-10 I21.0, I21.1, I21.2, I21.3), NSTEMI (ICD-10 I21.4), or unspecified (ICD-10 I21.9) Stroke admissions were differentiated into subtypes: ischemic stroke (ICD-10 I63), haemorrhagic stroke (ICD-10 I61, I64), transient ischemic attack, TIA (ICD-10 G45). To cover case fatality for acute MI (and for subtypes) and stroke, an in-hospital death was coded if the individual died in hospital. Age in years was used for descriptive statistics (Mean, SD) and in age categories of 60–79, 80–84, 85–89 and above 90 for the multivariate model. To calculate the mean age of the population, the age last entered into the database was used. Age was entered once a year into the database. Gender was coded male/female. Δ% refers to the number of incidences 2020–2021 in the whole population compared to the number of incidence 2015–2019, respectively. These numbers were weighted for the length of the respective period (2015–2019; 2020–2021) in months. Case fatality risks were calculated as the proportion of those who died in hospital out of those cases hospitalised with the respective diagnosis MI or stroke (where Incidence Risk Ratios [IRR] were used to compare 2020–2021 against 2015–2019). Sensitivity analyses with additional covariates (i.e., care level, heart failure, atrial fibrillation, pulmonary embolism, hypertension, and diabetes) albeit shorter observation period have been conducted (see Appendix for more details).

### Statistical analysis

For descriptive statistics, counts and percentages of hospital admissions and in-hospital deaths were presented. Differences in the number of incidences (Δ%) for each type of incidence were calculated for 2022–2021 separately and compared with the number of incidences in the five pre-pandemic years (2015–2019). These numbers were weighted to account for differences in the lengths of the periods.

$$ \Delta \% = \left( {\left( {\frac{Incidences\,2020{-}2021}{{{{Incidences\,2015{-}2019} \mathord{\left/ {\vphantom {{Incidences\,2015{-}2019} {3.75}}} \right. \kern-0pt} {3.75}}}}} \right) - 1} \right) \times 100 $$

Formula: Differences in the number of incidences.

IRR were obtained through adjusted Poisson generalized linear models with a log-link function. Models were employed for each group of incidences (hospitalisation/death) among types of incidences (MI/stroke) and corresponding subtypes (STEMI/NSTEMI; ischemic /haemorrhagic stroke/TIA, respectively) separately. We calculated the weekly rates of incidences for each type of incidence among age by gender groups and adjusted for effects of group size using a log-normal offset of the group size. Group size was specified by the share of LTCF population for hospitalisation and by the share in hospitalisations for death. Therefore, IRR represents the risk of hospitalisation among all LTCF residents and the risk of death among those who have been hospitalised. The exponential coefficients for 2020–2021 (ref. 2015–2019) in each regression model represent the corresponding IRR. Confidence Intervals (CI95%) were calculated using heteroscedasticity-consistent standard errors. Additionally, monthly incidence rates for STEMI/NSTEMI and ischemic stroke/haemorrhagic stroke in hospitalisation incidences were illustrated using Locally Estimated Scatterplot Smoothing Curves (LOESS) with a cubic B-spline function. All analyses were performed in the R environment for statistical computing (R version 4.1.1.) and RStudio (Version 1.3.959) including packages ‘stats’, ‘sandwich’, and ‘deltamethod’.

## Results

**Figure 1:**
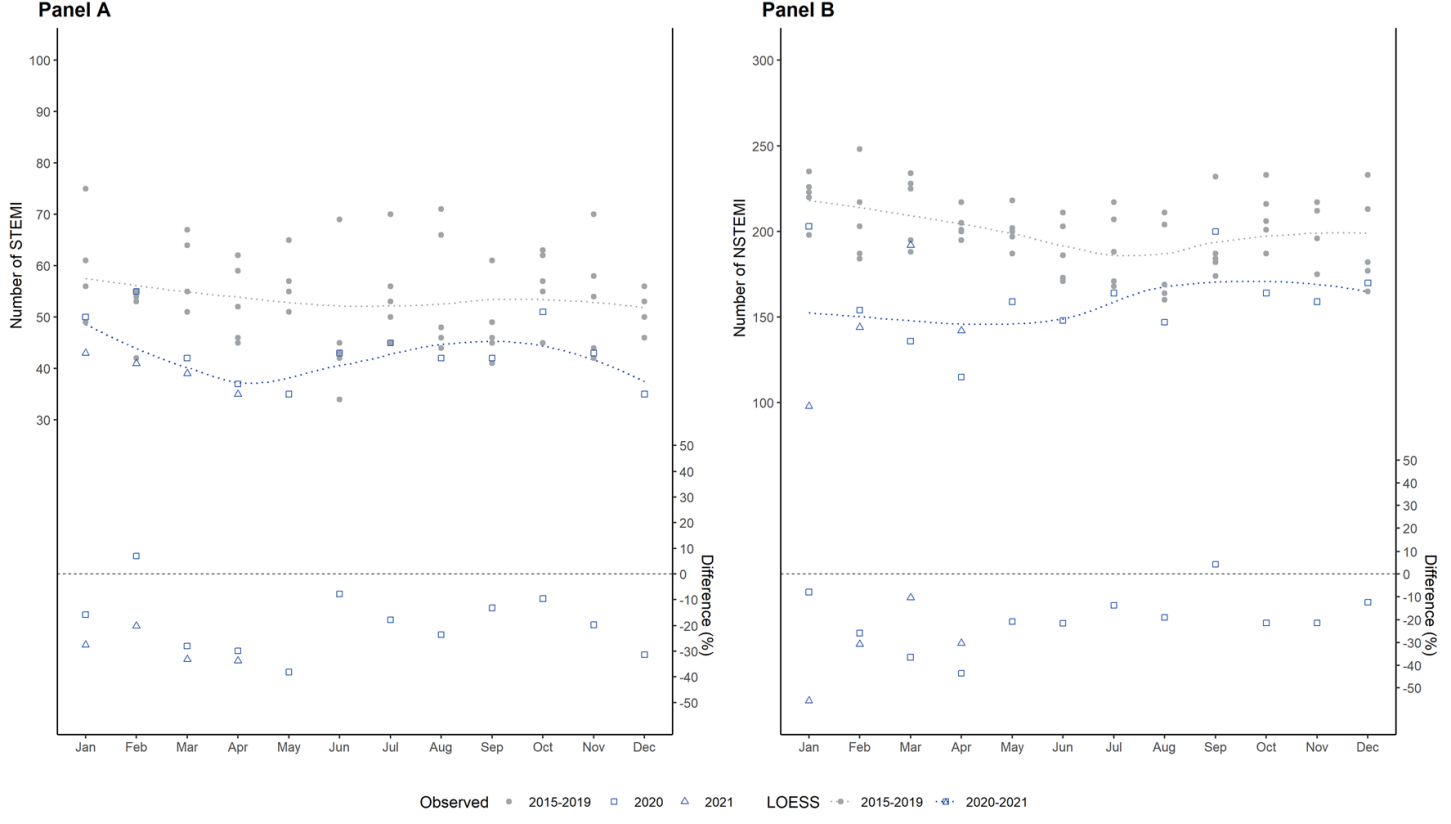
Number of acute myocardial infarction for STEMI (Panel **A**) and NSTEMI (Panel **B**) by months (crude numbers without denominator). Blue dots refer to the included months during the COVID-19 pandemic (2020–2021). Grey dots refer to the mean number of admissions in the five years prior to the pandemic (2015–2019). The line was estimated using Locally Estimated Scatterplot Smoothing Curves (LOESS) with a cubic B-spline function. The difference in percent between pandemic and pre-pandemic years is shown in the lower part of the figure.

From an overall dataset of 1,140,139 LTCF residents aged 60 years and older (Resident for at least one week during 2015 until April 2021; Weekly number of residents: Mean 348,116.8 [SD 7057.2]), a total of 15,886 MI admissions with a mean age of 83.8 years (SD = 8.0) were recorded during 2015–2019 of which 63.9% were women. In 2022–2021, 56.9% had hypertension, 27.5% heart failure, and 24.1% atrial fibrillation; this was 66.4% for hypertension, 30.9% for heart failure, and 25.6% for atrial fibrillation in 2016–2019 (see Appendix for more details). During the pandemic (2020–2021), 3227 MI hospital admissions were recorded (mean age 83.9, SD = 8.4, 61.6% were women). Regarding MI subtypes, about three out of four were NSTEMI in 2015–2019 (n = 12,020; 75.7%) with comparable figures in 2020–2021 (n = 2495; 76.05%; see Table 1). The average length of hospital stay for MI was 8.9 days. MI in-hospital deaths ranged from n = 4704 (29.6%) in 2015–2019 to n = 884 (26.9%) in 2020–2021. Mean age of LTCF patients with MI was 83.8 (SD = 8.0; 63.9% women) in 2015–2019, and 83.9 (SD = 8.4; 61.5% women) in 2020–2021. The trajectories of MI hospital admissions across months are displayed in Fig. 1 for STEMI (Panel A) and NSTEMI (Panel B), where declines in admission rates could be detected for NSTEMI (April 2022 and January 2021) and for STEMI (April till May 2020 and January till April 2021).

**Table 1:** Acute myocardial infarction and stroke admission and fatalities by year of observation.

	2015–2019			2020–2021^a^		
	N (%)	Age M (SD)	Female %	N (%)	Age M (SD)	Female %
MI hospital admissions						
Total	15,886 (82.9%)	83.8 (8.0)	63.9	3283 (17.1%)	83.9 (8.4)	61.5
NSTEMI	12,020 (75.7%)	83.8 (7.9)	62.7	2495 (76.0%)	83.8 (8.4)	60.4
STEMI	3222 (20.3%)	83.6 (8.3)	67.5	678 (20.7%)	84.1 (8.5)	63.3
Unspecified	644 (4.1%)	85.2 (8.0)	68.3	110 (3.4%)	85.3 (8.5)	75.5
MI-related deaths						
Total	4704 (29.6%)	84.8 (7.8)	65.3	884 (26.9%)	85.3 (7.9)	61.2
NSTEMI	2769 (23.0%)	84.8 (7.8)	63.6	517 (20.7%)	85.2 (7.8)	57.1
STEMI	1467 (45.5%)	84.5 (7.9)	67.9	292 (43.1%)	85.4 (8.2)	64.4
Stroke hospital admissions						
Total	60,297 (81.5%)	84.6 (7.8)	72.1	13,656 (18.5%)	84.8 (8.0)	70.1
Ischemic stroke	40,860 (67.8%)	84.9 (7.7)	72.9	9470 (69.3%)	85.0 (8.0)	71.0
Haemorrhagic stroke	5824 (9.7%)	83.6 (8.0)	69.7	1256 (9.2%)	83.7 (8.2)	67.3
TIA	13,613 (22.6%)	84.2 (7.8)	70.8	2930 (21.5%)	84.7 (7.9)	68.6
Stroke-related deaths						
Total	11,543 (19.1%)	86.0 (7.3)	74.6%	2629 (19.3%)	86.4 (7.5)	73.3
Ischemic stroke	8633 (21.1%)	86.5 (7.2)	76.2	1966 (20.8%)	86.9 (7.3)	74.5
Haemorrhagic stroke	2679 (46.0%)	84.5 (7.7)	70.1	609 (48.5%)	84.7 (7.8)	69.5
TIA	231 (1.7%)	85.5 (7.1)	66.2	54 (1.8%)	88.2 (6.5)	72.2

MI = Myocardial infarction; STEMI = ST-segment elevation myocardial infarctions; NSTEMI = non-ST-segment elevation myocardial infarction; TIA = transient ischemic attack.

^a^2020–2021 includes cases with an admission date between 01/01/2020 and 04/30/2021 and a discharge date up to 06/30/2021.

**Figure 2:**
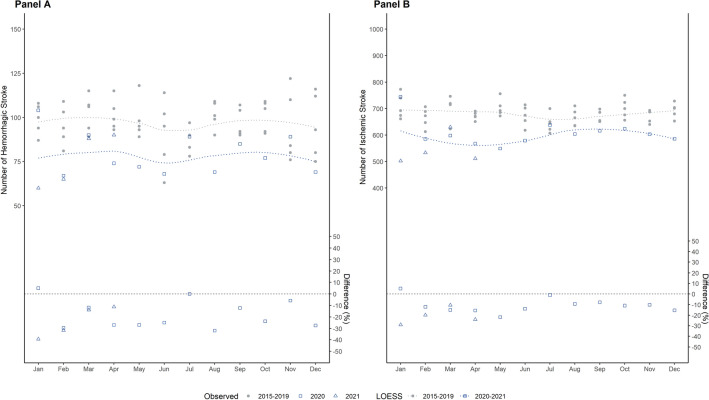
Number of stroke hospital admissions for haemorrhagic (Panel **A**) and ischemic (Panel **B**) stroke by months (crude numbers without denominator). Blue dots refer to the included months during the COVID-19 pandemic (2020–2021). Grey dots refer to the mean number of admissions in the five years prior to the pandemic (2015–2019). The line was estimated using Locally Estimated Scatterplot Smoothing Curves (LOESS) with a cubic B-spline function. The difference in percent between pandemic and pre-pandemic years is shown in the lower part of the figure.

Concerning stroke admissions, we recorded n = 60,297 strokes in 2015–2019 in patients with a mean age of 84.6 (SD = 7.8) years, and n = 15,056 (mean age 84.8, SD = 8.0; 70.2% were women) in the months of the pandemic. Those were, on average, 84.8 (SD = 8.0; 72.1% female) years of age in 2015–2019 and 84.9 (SD = 8.0; 70.1% women) in 2020–2021. Two-thirds were ischemic stroke with n = 40,860 (67.8%) in 2015–2019 and n = 9470 (69.3%) in 2020–2021. Stroke deaths ranged from 19.1% (n = 11,543) in 2015–2019 to 19.3% (n = 2629) in 2020–2021. The trajectories of stroke hospital admissions across months are displayed Fig. 2. Lower rates for stroke were found through the waves of the pandemic compared to the previous years. The average length of stay for stroke was 9.8 days in our study.

There was a decline in MI admission during the pandemic, derived from the multivariate model, compared to the previous years by 22.5% (IRR = 0.68 [CI95% 0.65–0.72]; < 0.001) (Table 2). This decline was more pronounced in NSTEMI with a decline of 22.2%; (IRR = 0.68 [CI95% 0.65–0.72]; < 0.001) than in STEMI (21.1%; IRR = 0.69 [CI95% 0.63–0.76; < 0.001). The MI case fatality was not significantly different in 2020–2021 (IRR = 0.97 [CI95% 0.92–1.02]; *p* = 0.249) compared with the prior years of observation.

**Table 2:** Estimated acute myocardial infarction and stroke admission and case fatality rates among LTCF residents.

	2020–2021^a^ versus 2015–2019	
	Change in admission and deaths	Case fatality rate
	Δ%	IRR (CI; *p*)
MI hospital admissions		
MI	− 22.5	0.68 (0.65–0.72; < 0.001)
STEMI	− 21.1	0.69 (0.63–0.76; < 0.001)
NSTEMI	− 22.2	0.68 (0.65–0.72; < 0.001)
MI-related in-hospital deaths		
MI	− 29.5	0.97 (0.92–1.02; 0.207)
STEMI	− 25.4	1.00 (0.94–1.08; 0.922)
NSTEMI	− 30.0	0.99 (0.93–1.06; 0.822)
Stroke hospital admissions		
Stoke	− 15.1	0.75 (0.72–0.78; < 0.001)
Ischemic	− 13.1	0.76 (0.73–0.80; < 0.001)
Haemorrhagic	− 19.1	0.71 (0.67–0.77; < 0.001)
TIA	− 19.3	0.71 (0.68–0.75; < 0.001)
Stroke-related in-hospital deaths		
Stroke	− 14.6	1.00 (0.96–1.04; 0.939)
Ischemic	− 14.6	0.99 (0.95–1.03; 0.654)
Haemorrhagic	− 14.8	1.09 (1.03–1.15; 0.002)
TIA	− 12.3	1.10 (0.97–1.25; 0.151)

MI = Myocardial infarction; STEMI = ST-segment elevation myocardial infarctions; NSTEMI = non-ST-segment elevation myocardial infarction; TIA = transient ischemic attack; Δ% = Difference in the number of incidences (admissions and death respectively) in 2015–2019 in the whole population of residents form long-term care facilities; IRR = incidence rate ratio for admissions/incidence risk ratio for MI and stroke in-hospital deaths among these residents who have been hospitalized due to MI or stroke; CI95% = 95% confidence interval; *p* = *p*-value; Poisson regression controlled for gender, age and population-group-size.

^a^2020–2021 includes cases with an admission date between 01/01/2020 and 04/30/2021 and a discharge date up to 06/30/2021.

Likewise, the stroke admission rates of LTCF residents dropped by 15.1% (IRR = 0.75 [0.72–0.78]; *p* < 0.001) in 2020–2021. Stroke fatality rates remained stable for overall stroke with IRR = 1.00 ([0.96–1.04]; *p* = 0.457) in 2020–2021. For stroke subtypes, there was a higher case fatality risk for haemorrhagic stroke (IRR = 1.09 [CI95% 1.03–1.15]; *p* = 0.002) in 2020–2021 than in the previous years. Other subtypes remained comparable across years (all *p* > 0.05).

## Discussion

Our primary objective was to investigate MI and stroke hospital admissions and deaths in residents of LTCF during the COVID-19 pandemic. We found a substantial decline in MI admissions by 22.5% during the pandemic in 2020–2021, compared with the years before the pandemic. These figures are lower than findings from a meta-analysis in the general population, where eleven studies found a decrease in admissions due to acute coronary events between 40 and 50%. Likewise, De Rosa et al. found a 48.4% reduction in admissions for MI compared with the period prior to the pandemic^15^. In our study, this comparable in NSTEMI with a decline of 22.2% in 2020–2021 and in STEMI (21.1%). The meta-analytical findings showed in four studies a greater decrease in NSTEMI compared to STEMI, while there was one study that did not find differences between MI subtypes^8^. A German study on the general population found MI rates declined by 44% during the lockdown in both STEMI and NSTEMI patients independently of gender and age^12^. Finally, an English study on LTCF residents found a substantial decrease during the first wave of the pandemic, with emergency admissions decreased by 29% for acute coronary syndromes^14^.

In the present study, stroke admission rates for residents of LTCF dropped by 15.1% in 2020–2021. Data from residents of English LTCF indicate a decrease in emergency admissions by 25% for stroke^14^, which is higher than our findings for stroke. Moreover, we found higher case fatality rates for haemorrhagic stroke in 2020–2021 only, while the figures for hospital admission rates for all other types of stroke did not change during the pandemic.

MI-related in-hospital deaths decreased by 29.5% in 2020–2021 in our study as a consequence of the reduced admission rates. However, the case fatality rates (i.e., deaths among those hospitalised) remained largely on the same level than in the years before. Stroke fatality rates decreased by 14.6% in 2020–2021. In the meta-analysis by Kiss et al. with patients from the general population, five studies showed a decrease in stroke admissions between 12 and 40%^8^. Concerning CVD deaths, Kiss et al. found four studies that reported higher death rates during the pandemic^8^.

Our study has strengths and limitations. The dataset used includes about half of all publicly insured LTCF residents in Germany, providing valid in-hospital data for MI and stroke admissions and mortality. Further, while there are some studies on the admission rates in the general population, there is a massive lack of data on older adults from LTCF; thus, filling this research gap is a major strength of the present study.

There are limitations that need to be considered. First, there could be slight distortion of frequencies due to hospital cases that were not completed in 2021. However, the impact is very small and only in the last included weeks of 2021. Second, although insurance claims data are comprehensive and selective inclusion or dropout of participants are not present in the data, clinical information about CVD is sparse. However, the available information was sufficient to answer our research questions. Due to a restricted period of data available, we decided to include care and disease-related covariates in sensitivity analyses only. This limits the main results partly, which are not fully adjusted. In future studies, routine data should be complemented by cohort studies based on primary data, if the aim is to investigate clinical mechanisms of decreased submission rates. Third, we were not able to distinguish older people living in LTCF from those living in nursing homes. This was because we had no direct marker to capture those older adults living in nursing homes, but included residents living in LTCF over 60 years of age or older. Therefore, older people with disability living in a LTCF, but not from nursing homes, are potentially in our sample as well. However, these number will be very small. Finally, although we drew on a large dataset covering all regions of the country the data is not representative for all LTCF residents in regard to age, gender and co-morbidities relevant to MI and stroke incidents as the share of the dataset is 49% only. However, AOK insures people with a high multimorbidity burden and deviation are more likely into the direction of people with poor health than to the healthier ones.

While the generally time-stable case fatality rates across years may indicate constant high-quality in-hospital treatment of MI and stroke patients during the pandemic as well as no major systematic selection processes in the patients being hospitalised, we found slightly higher case fatality rates for haemorrhagic stroke during the pandemic than in the years before. This finding could reflect, again, either changes in the treatment during the first wave of the pandemic or the possibility that only more severe cases were hospitalised during the pandemic, which could have increased the case fatality rate.

The decline in admission rates among residents from LTCF may be due to potential changes of hospitals’ admission and emergency strategies or changes in the referral strategies and behaviours within the LTCF, hesitating to send residents to the hospitals; although we did not find published evidence in the literature for these hypotheses. Nonetheless, this reduction is highly problematic, since, in principle, MI and stroke constitute indication for inpatient emergency hospital submission. Of course, exceptions exist, for instance in those people at the end of life with a respective living will / statement. Potential reasons for that patterns include fewer MI and strokes occurred or residents with an MI or stroke were less likely to be admitted to the hospital. The latter could be because it was less often observed. While reduction rates in admissions from LTCF are comparable or even slightly lower than in the general population in other countries (only one other study in the systematic review of Kiss et al. was from Germany^16^), the often very old and multimorbid LTCF residents constitute a fraction of the most vulnerable MI and stroke patients, where any delay in treatment needs to be avoided. The only other available data from the UK of LTC admissions to hospital by Grimm et al. found large reductions during the pandemic for elective admissions as well as avoidable emergency admissions including urinary tract infections, which may be subject of prioritised primary care treatment during the pandemic^14^. In addition, however, Grimm et al. found a reduction in acute admissions such as MI and stroke, which should be a matter of concern as these might even relate to increased death rates during the pandemic. Preventive control guidelines for LTCF should include establishing relationships with acute hospitals, including admission policies to hospitals in times of major crises to guarantee optimal emergency care^17^.

We observed substantially reduced rates of MI and stroke hospital admission rates in residents of LTCF during the pandemic. The numbers calling for measures that assure optimal acute care of residents of LTCF for future pandemics and crises.

## Supplementary Information

Supplementary Information.

## Data Availability

Data and materials are available from the corresponding author on reasonable request. The data used in this study cannot be made available in the manuscript, the supplementary files, or in a public repository due to German data protection laws (Bundesdatenschutzgesetz). Therefore, data are stored on a secure drive in the Wissenschaftliches Institut der AOK to facilitate replication of the results. Generally, access to data of statutory health insurance funds for research purposes is possible only under the conditions defined in the German Social Law (SGB V § 287). Requests for data access can be sent as a formal proposal, specifying the recipient and purpose of the data transfer, to the appropriate data protection agency. Access to the data used in this study can only be provided to external parties under the conditions of the cooperation contract of this research project and after written approval by the AOK. For assistance in obtaining access to the data, please contact wido@wido.bv.aok.de.

## References

[1] Kohl R, Schwinger A, Jürchott K (2022). Mortality among hospitalized nursing home residents with COVID-19. Deutsch. Arztebl. Int..

[2] Chew NW, Ow ZGW, Teo VXY (2021). The global effect of the COVID-19 pandemic on STEMI care: A systematic review and meta-analysis. Can. J. Cardiol..

[3] Koge J, Shiozawa M, Toyoda K (2021). Acute stroke care in the with-COVID-19 era: Experience at a comprehensive stroke center in Japan. Front. Neurol..

[4] Kristoffersen ES, Jahr SH, Thommessen B, Rønning OM (2020). Effect of COVID-19 pandemic on stroke admission rates in a Norwegian population. Acta Neurol. Scand..

[5] Henry TD, Kereiakes DJ (2022). The direct and indirect effects of the COVID-19 pandemic on cardiovascular disease throughout the world. Eur. Heart J..

[6] Bollmann A, Pellissier V, Hohenstein S (2021). Cumulative hospitalization deficit for cardiovascular disorders in Germany during the COVID-19 pandemic: Insights from the German-wide Helios hospital network. Eur. Heart J. Qual. Care Clin. Outcomes.

[7] Mariet A-S, Giroud M, Benzenine E (2021). Hospitalizations for stroke in France during the COVID-19 pandemic before, during, and after the national lockdown. Stroke.

[8] Kiss P, Carcel C, Hockham C, Peters SA (2021). The impact of the COVID-19 pandemic on the care and management of patients with acute cardiovascular disease: A systematic review. Eur. Heart J. Qual. Care Clin. Outcomes.

[9] König S, Ueberham L, Pellissier V (2021). Hospitalization deficit of in-and outpatient cases with cardiovascular diseases and utilization of cardiological interventions during the COVID-19 pandemic: Insights from the German-wide helios hospital network. Clin. Cardiol..

[10] Bersano A, Kraemer M, Touzé E (2020). Stroke care during the COVID-19 pandemic: Experience from three large European countries. Eur. J. Neurol..

[11] Ball S, Banerjee A, Berry C (2020). Monitoring indirect impact of COVID-19 pandemic on services for cardiovascular diseases in the UK. Heart.

[12] Schmitz T, Meisinger C, Kirchberger I (2021). Impact of COVID-19 pandemic lockdown on myocardial infarction care. Eur. J. Epidemiol..

[13] Bronskill, S. E., Maclagan, L. C., Maxwell, C. J. *et al.* in *JAMA Health Forum* e214599–e214599. (2022). 10.1001/jamahealthforum.2021.4599 PMC8903126 35977228

[14] Grimm F, Hodgson K, Brine R, Deeny SR (2020). Hospital admissions from care homes in England during the COVID-19 pandemic: A retrospective, cross-sectional analysis using linked administrative data. Int. J. Popul. Data Sci..

[15] De Rosa S, Spaccarotella C, Basso C (2020). Reduction of hospitalizations for myocardial infarction in Italy in the COVID-19 era. Eur. Heart J..

[16] Gitt A, Karcher A, Zahn R, Zeymer U (2020). Collateral damage of COVID-19-lockdown in Germany: Decline of NSTE-ACS admissions. Clin. Res. Cardiol..

[17] Calcaterra L, Cesari M, Lim WS (2022). Long-term care facilities (LTCFs) during the COVID-19 pandemic—Lessons from the Asian approach: A narrative review. J. Am. Med. Dir. Assoc..

