# Supplementary material for: Hospital admissions and deaths due to acute cardiovascular events during the COVID-19 pandemic in residents of long-term care facilities
Source: Sci Rep. 2023 May 26;13:8544. doi: 10.1038/s41598-023-35816-y (PMC10214327; doi:10.1038/s41598-023-35816-y)
Supplement: Supplementary file 1 — Supplementary Information. [file 41598_2023_35816_MOESM1_ESM.docx]

**Appendix:** Hospital admissions and deaths due to acute cardiovascular events during the COVID-19 pandemic in residents of long-term care facilities by Paul Gellert, Raphael Kohl, Kathrin Jürchott, Betty Noack, Christian Hering, Annabell Gangnus, Elisabeth Steinhagen-Thiessen, Wolfram J Herrmann, Adelheid Kuhlmey, and Antje Schwinger

**Appendix Table A1: Care dependency, co-morbidities and observation time by time period**

| **Variable,** | **Period** | | **p value** |
| --- | --- | --- | --- |
|  | **2016-2019**  mean (SD) | **2020-2021**  mean (SD) |  |
| Care dependency level | 3.5 (0.03) | 3.5 (0.01) | 0.541 |
| Heart failure (%) | 30.9 (1.7) | 27.5 (13.5) | 0.040 |
| Atrial fibrillation (%) | 25.6 (2.1) | 24.1 (11.8) | 0.017 |
| Pulmonary embolism (%) | 1.6 (0.2) | 1.7 (0.8) | 0.017 |
| Hypertension (%) | 66.4 (1.5) | 56.9 (27.9) | 0.070 |
| Diabetes (%) | 17.5 (0.2) | 14.7 (7.2) | 0.802 |
| Mean observation time (%) | 2.5 (0.7) | 3.7 (0.1) | <0.001 |

*Note:* p values refer to Wilcoxon rank sum exact test. Heart failure: I099, I110, I130, I132, I255, I42, I43, I50 ICD: C01A, C03, C07, C09A, C09B, C09C, C09D; Arterial fibrillation: I48; Pulmonary embolism: I26; Hypertension: I10, I119, I129, I139, I15 ICD: C02, C03, C07, C08, C09; Diabetes: E10, E11, E12, E13, E14 ICD: A10A, A10B, A10X. The German care levels refer to the need of care where 1 is low and 5 is high need for care.

**Appendix Table A2: Sensitivity Analysis – Estimated acute myocardial infarction and stroke admission and case fatality rates among long-time care facilities residents adjusted gender, age, care dependency level, heart failure, atrial fibrillation, pulmonary embolism, hypertension, diabetes and population-group-size**

|  | 2020-21 vs. 2016-19^a^ | |
| --- | --- | --- |
|  | Change in admission and deaths  Δ% | Case fatality rate  IRR (CI; p) |
| MI hospital admissions |  |  |
| MI | -38.0 | 0.80 (0.77-0.84; <0.001) |
| STEMI | -17.7 | 0.92 (0.86-0.99; <0.001) |
| NSTEMI | -20.7 | 0.80 (0.77-0.84; <0.001) |
| MI-related in-hospital deaths |  |  |
| MI | -26.1 | 0.97 (0.92-1.03; 0.477) |
| STEMI | -20.9 | 1.01 (0.94-1.08; 0.931) |
| NSTEMI | -27.5 | 0.99 (0.93-1.05; 0.822) |
| Stroke hospital admissions |  |  |
| Stoke | -31.2 | 0.86 (0.84-0.88; <0.001) |
| Ischemic | -13.0 | 0.87 (0.85-0.89; <0.001) |
| Haemorrhagic | -16.9 | 0.86 (0.81-0.91; <0.001) |
| TIA | -18.2 | 0.82 (0.79-0.86; <0.001) |
| Stroke-related in-hospital deaths |  |  |
| Stroke | -13.9 | 1.00 (0.96-1.04; 0.967) |
| Ischemic | -14.0 | 0.99 (0.95-1.04; 0.835) |
| Haemorrhagic | -13.4 | 1.07 (1.01-1.13; 0.135) |
| TIA | -14.3 | 1.04 (0.92-1.20; 0.765) |
| *Note:* MI = Myocardial infarction; STEMI = ST-segment elevation myocardial infarctions; NSTEMI = non-ST-segment elevation myocardial infarction; TIA = transient ischemic attack; Δ% = Difference in the number of incidences (admissions and death respectively) in 2016-2019 in the whole population of residents form long-term care facilities; IRR = Incidence Rate Ratio for admissions / Incidence Risk Ratio for MI and stroke in-hospital deaths among these residents who have been hospitalized due to MI or stroke; CI95% = 95% confidence interval; p = p-value; Poisson regression controlled for gender, age, care dependency level, heart failure, atrial fibrillation, pulmonary embolism, hypertension, diabetes and population-group-size; ^a^2020-2021 includes cases with an admission date between 01/01/2020 and 04/30/2021 and a discharge date up to 06/30/2021. | | |
